# Comparison of phosphor screen autoradiography and micro-pattern gas detector based autoradiography for the porosity of altered rocks

**DOI:** 10.1038/s41598-020-65791-7

**Published:** 2020-06-11

**Authors:** C. Delayre, J. Sammaljärvi, S. Billon, E. Muuri, P. Sardini, M. Siitari-Kauppi

**Affiliations:** 1 Université de Poitiers, UMR CNRS 7285 IC2MP / HYDRASA, rue Michel Brunet, 86022 Poitiers Cedex, France; 2 Department of Chemistry, University of Helsinki, 00014 Helsinki, Finland; 3 ERM company, 4 rue Carol Heitz, 86000 Poitiers, France

**Keywords:** Geology, Mineralogy, Petrology

## Abstract

This study aims to further develop the ^14^C-PMMA porosity calculation method with a novel autoradiography technique, the Micro-pattern gas detector autoradiography (MPGDA). In this study, the MPGDA is compared with phosphor screen autoradiography (SPA). A set of rock samples from Martinique Island exhibiting a large range of connected porosities was used to validate the MPGDA method. Calculated porosities were found to be in agreement with ones from the SPA and the triple-weight method (TW). The filmless nature of MPGDA as well as straightforward determination of C-14 radioactivity from the source rock makes the porosity calculation less uncertain. The real-time visualization of radioactivity from C-14 beta emissions by MPGDA is a noticeable improvement in comparison to SPA.

## Introduction

Autoradiography has demonstrated its adaptability and reliability in several research areas. With regard to the development of autoradiographic techniques the very first autoradiograph was obtained by Becquerel in 1896, through exposition of an emulsion of silver halide crystals in contact with radioactive material^1^. Autoradiographic techniques allow the production of spatial distribution of radioactivity in the material; the sample itself being the source of the radioactivity either as occurring naturally in the material or as artificially added radioactivity. In the case of geological samples, ^238^U, ^232^Th and ^40^K bearing minerals are the main sources of natural radioactivity^1^. The radioactive source may also be in the form of an artificial radioactive marker, such as ^3^H or ^14^C labelled methylmethacrylate (MMA)^2–4^. Investigated samples are impregnated with the radiolabeled tracer and fixed in place via polymerization of the marked MMA in the connected pore network of rock samples. Thus, the image of the connected pore network can be produced by exposing the impregnated sample surface on a photographic film. This brief description corresponds to the ^14^C polymethylmethacrylate (^14^C-PMMA) autoradiography method^2–4^ and will be used for porosity analysis in this work.

In the field of nuclear waste management, the ^14^C-PMMA method has been successfully applied to investigate the connected pore network of geological materials, radioelements diffusion within rock matrices and relations between the porosity and mineralogy of rocks^5–8^. In the biological field, injecting the radioligands into living tissues allows to map the anatomical location of specific receptors by autoradiographic techniques. For instance, Larsson & Ullberg^9^ introduced a new autoradiographic technique, the whole body macro autoradiography (WBA), allowing to study the distribution of ^35^S-labelled penicillin in a rat using entire frozen sections of them after injection of radiolabeled tracer. In drug distribution studies, Young and Kuhar^10^ were able to localize opioid receptors in a rat brain by ^14^C, ^3^H, ^125^I and ^35^S, and more recently, Fang *et al*.^11^ studied the effect of cocaine, ketamine and methamphetamine on dopamine terminals in the striatum of rats using ^18^F. These molecules are known to play an important role in drug taking and drug seeking behaviors. Nowadays, and after several improvements, WBA has become a standard technique for studying the distribution of drugs in tissues^12^.

The traditional autoradiography technique, film autoradiography (FA) produces a blackening of the photographic film resulting from the reduction of Ag^+^ ions within a silver-halide grain of the film emulsion when exposing the sample on the photographic film^13,14^. The film autoradiographs can be digitized by scanning them with CCD camera or a desktop scanner to produce 8-bit or 16-bit depth digital images^2,15^. Over the past few decades, further advances have been made to further develop autoradiography techniques, mainly for beta particles detection in biological field. Four kinds of technologies have been developed: (1) storage phosphor autoradiography (SPA) using phosphor imaging plates (IP) composed of alkali halide material (e.g. BaFBr:Eu^2+^)^14–16^, (2) gaseous detector allowing to count electronic charge induced by radiation-gas interaction (ionization)^17–21^, (3) semi-conductor sensor able to convert radiation or optical signal into electric signal such as CCD (Charged Coupled Device) or CMOS (Complementary Metal Oxide Semiconductor)^22–25^, and (4) scintillation based detector as the BetaIMAGER™ ^D^FINE^26^, where radiations are converted into light before to be detected with sensor coupled with an amplification system^27–29^. The last three aforementioned technologies are filmless and real time autoradiography.

During the 1980s, SPA technique was developed using phosphor imaging plates (IP) composed of alkali halide material (e.g. BaFBr:Eu^2+^). SPA is based on a photo stimulated luminescence mechanism (PSL), which consists of the emission of photons resulting from the recombination of electrons (trapped in Br vacancies) with Eu^3+^ after stimulation with red laser beam^14,30^. It has several advantages compared to FA as reported by Sardini *et al*.^15^. According to the authors, one advantage of using SPA is the possibility to reuse the phosphor imaging plates after erasing the latent image by subsequent exposure to light. Moreover, the sensitivity of SPA has been reported to be 60 to100 times higher than that of FA^14^. SPA has a linear dynamic range covering 4–5 orders of magnitude while FA has a linear response of only two orders of magnitude^1^. However, in SPA technique the long exposures (more than 7 days) are not possible because of the fading process that exist in phosphor imaging plates^14^.

The gaseous detector BeaQuant is a new autoradiography technique that couples a micro patterned gaseous detector (MPGD) and parallel ionization multiplier (PIM) device^19,30–32^. This system allows count to-count detection of radiation emitted from the sample. Therefore, the MPGDA offers a linear response of 5 decades in detection of radiations in the limits of the capacity of the electronic counting device. Real-time visualization can be displayed enabling the user to stop the acquisition at any moment and thus optimize the time of acquisition. The resolution is about 20 µm for ^3^H. In the case of natural radioactive samples, such as rocks composed of uranium-bearing minerals, separation of alpha emitting nuclides from beta emitters is possible^33^. It has to be noted that this is a valuable advantage compared to SPA since the latter cannot separate alpha emitters from electrons. It is particularly of a great interest for geologists who use disequilibrium in uranium decay chains as a probe to date rocks^34^. The first study to apply MGPDA for quantitative mapping of alpha emissions from rock sections has been performed by Sardini *et al*.^33^.

As a summary Table 1 presents the different characters for the autoradiographic techniques discussed in this work.

**Table 1:** Characters of film, storage phosphor, micro-pattern gas detector and electronic autoradiographies.

	FA	SPA	MPGDA	EA-Scintillation^28^
	Film Autoradiography	Storage Phospor Autoradiography	Micro-pattern gas detector Autoradiography	Scintillation Autoradiography
Activity	through intensity treated as optical densities	through photo stimulated luminescence values	counts/second/area	counts/second/area
Linearity	2 decades	5 decades	5 decades	5 decades
Real Time	no	no	yes	possible
Numerical	by desktop scanning	by laser scanning	yes	yes
Field of view	15*20 cm^2^	15*20 cm^2^	8*12 cm^2^ or more	2.5*2.5 mm^2^
influence by gamma or X-rays	yes	no	no	yes
other background sources	any fluorescence emissions	any fluorescence emissions	no	no
alpha and beta separation	no	no	yes	possible
main other ADVANTAGES	cheap	reusable films	times are recorded	no use of electric field

The reasons to do this study were: (1) to improve the ^14^C-PMMA autoradiographic method for the porosity determination of hard materials, (2) to improve the sensitivity of the ^14^C beta emissions detection, (3) to get rid of the background uncertainties which are present in the Röntgen film and SPA autoradiography. Here, SPA and MGPDA techniques were compared qualitatively and quantitatively in the context of ^14^C-PMMA method. The detector efficiency of the BeaQuant system was investigated using Monte Carlo simulations. The porosity of several volcanic rock samples (Martinique island, French Caribbean) presenting a broad range of porosity were determined with TW, SPA and MGPDA techniques.

## Results and Discussion

**Figure 1:**
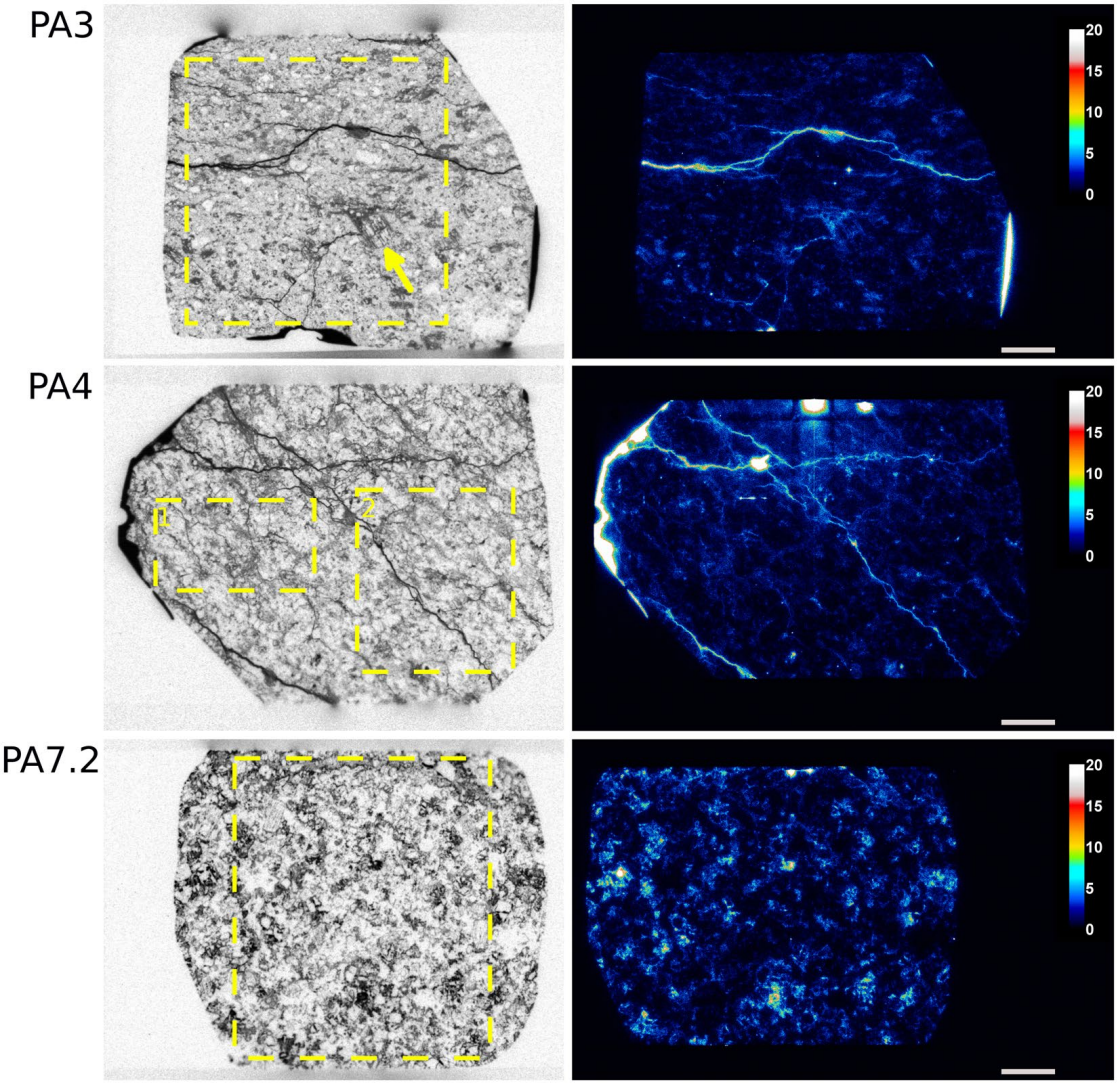
SPA (left) and MPGDA (right) of samples PA3; PA4 and PA7.2 of the series Petite-Anse. Scale bar (white) is 0.5 cm. The unit of color scales for MPGDA is cps/pixels. Porosity calculation are performed within yellow dotted areas. Dark arrow shows hornblende crystal (PA3).

**Figure 2:**
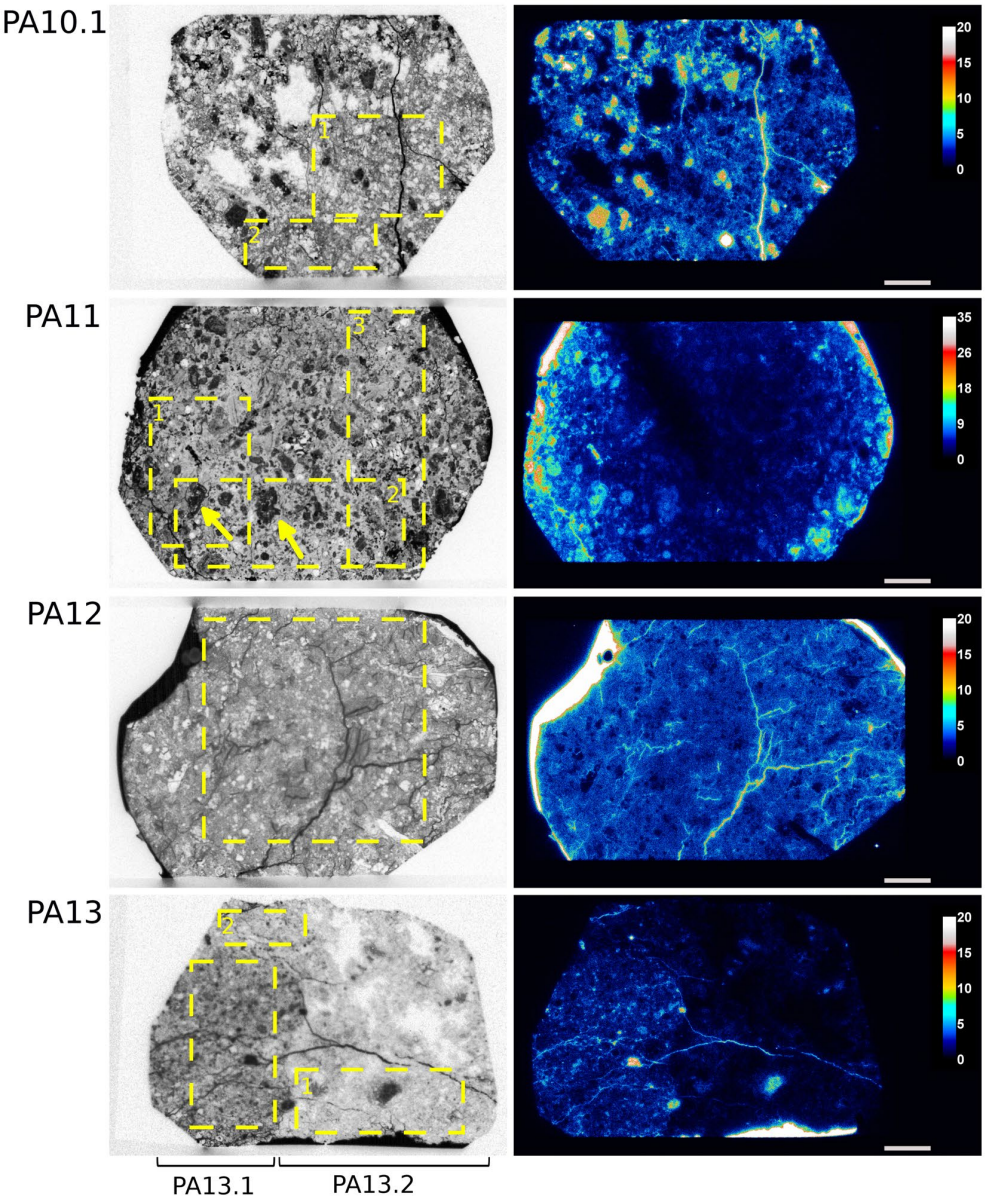
SPA (left) and MPGDA (right) of samples PA10.1; PA11; PA12 and PA13 of the series Petite-Anse. Scale bar (white) is 0.5 cm. The unit of color scales for MPGDA is cps/pixels. Porosity calculations are performed within yellow dotted areas. Yellow arrows shows plagioclase crystals (PA11). PA13.2 corresponds to the wall rock of sample PA13.

**Figure 3:**
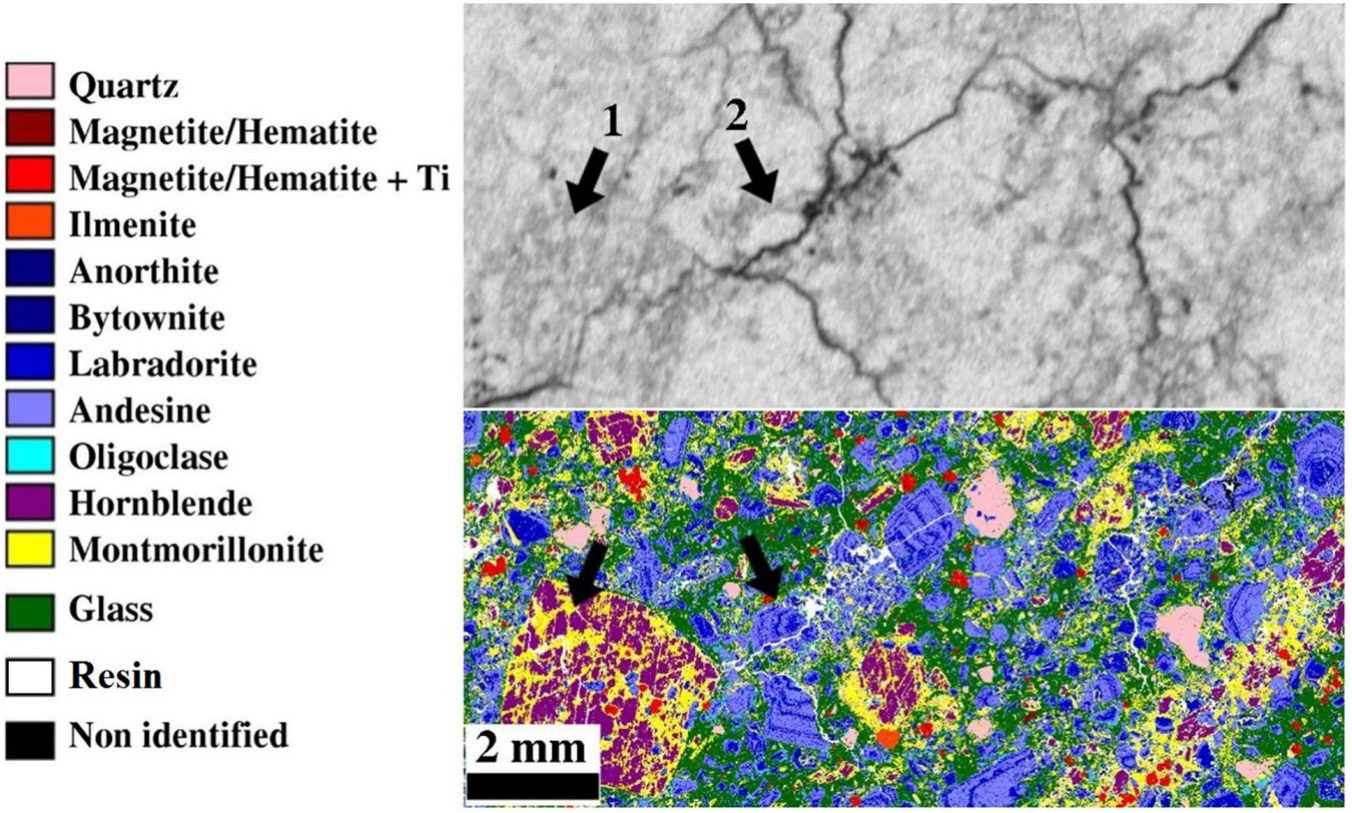
The SPA autoradiograph of sample PA4 and the corresponding mineral map by QEMSCAN analyses.

Figures 1 and 2 present the autoradiographs of studied samples obtained with SPA and MPGDA. Some amount of pure ^14^C-PMMA resin can be observed on the side of samples. They are seen as darkest and white features on SPA and MPGDA, respectively. Furthermore, the connected pore structure of the impregnated samples is shown in the SPA and MPGDA images. Darker the shade higher the porosity on the SPA images, whereas for MPGDA, colors from blue through red to white shows the various spatial porosities in the samples. Several features can be observed regarding the nature of the rock series. Millimetric scale minerals such as hornblende, plagioclases and quartz can be clearly identified. For example, in sample PA3, a crystal of hornblende of about 3 mm presents parallel cleavages (due to longitudinal section) where the resin has penetrated, facilitated by the argillic alteration (Fig. 1). In the sample PA11, because of the advanced stage of alteration, it is possible to distinguish oscillatory zoning in “ghost plagioclases” (see arrows on the Fig. 2). Such thin structures can also be identified on MPGDA but with less definition as compared to SPA. This indicates that SPA has better resolution than MPGDA. Several samples (i.e. PA3; PA4; PA10.1; PA12 and PA13) show fractures which are clearly revealed by both techniques. Impregnation was possibly incomplete in parts of the samples PA13 and PA 10.1 which is probably due to altered minerals that are difficult to dry or are swelling clays. Up to 22% of montmorillonite was observed in sample PA11^35^ (Figs. 2 and 3). Mineral maps can be used to study the correlation between the mineralogy and their specific porosity. Such correlation has been visually estimated by using mineral map obtained from Quemscan system^35^. An example of such correlation is presented in Fig. 3.

In MPGDA the roughness of the surface or the dust particles on the sample surface may cause artefacts that might be seen as bright spots on the MPGDA image. This is the case for sample PA4 where a “hot spot” is clearly visible (Fig. 1). This was also found in the work of Voutilainen *et al*. where the porosities of granites from Olkiluoto, Finland were studied^36^. Another issue has to be noted for the MPGDA where a “dead zone” crosses the PA11 sample. It is attributed to a technical problem of the BeaQuant system during the acquisition since no porosity gradient was found from the edge to the core sample. For these samples, porosity calculations were performed excluding these anomalous areas.

**Figure 4:**
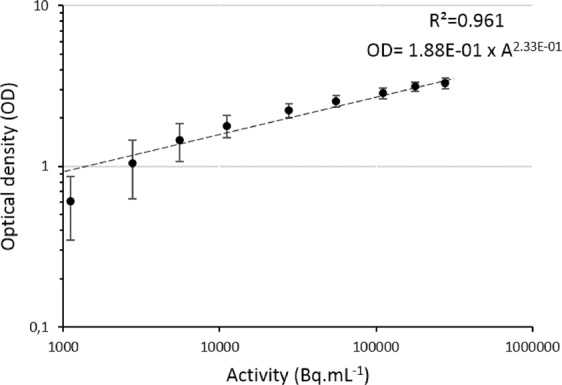
Calibration of SPA using ^14^C-PMMA standards (1-280 kBq.mL−1). Error bars represent the standard deviation of optical densities obtained for each standard.

**Figure 5:**
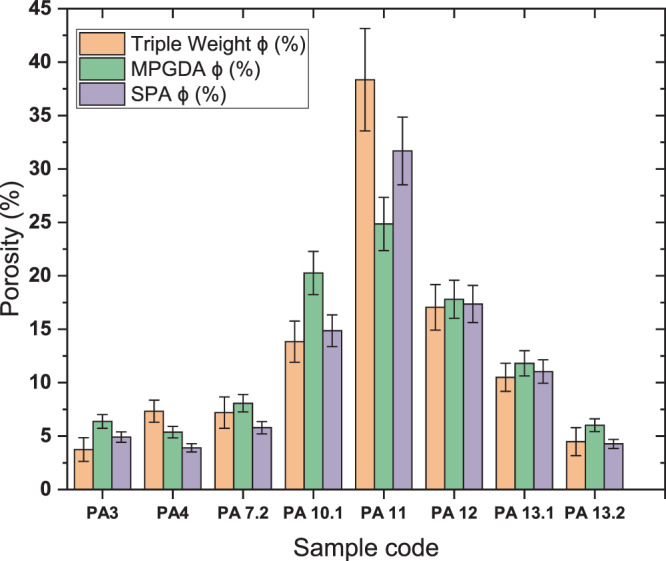
Comparison of the porosity values measured with TW, MPGDA and SPA methods.

**Figure 6:**
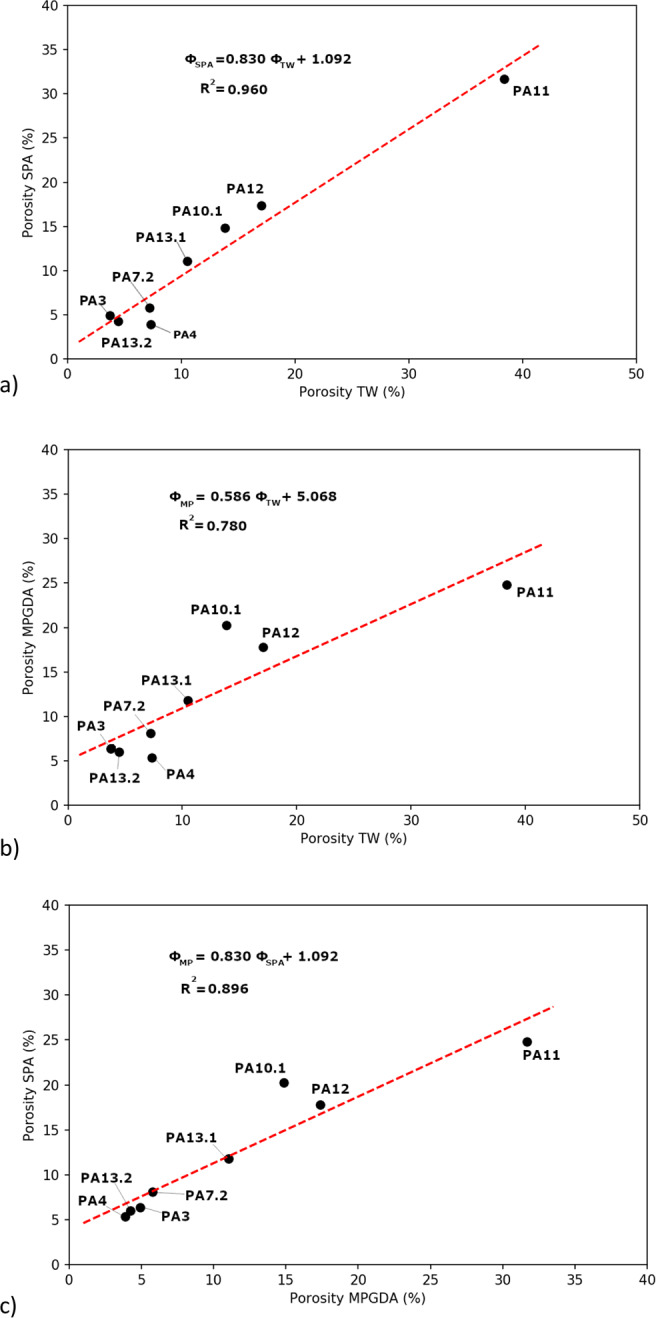
Porosity of samples calculated from SPA (**a**) and MPGDA (**b**) versus bulk porosity measurements (TW) and comparison between porosities calculated from MPGDA and SPA (**c**).

Figure 4 presents the results of the calibration step for SPA. During the fitting process, the two parameters *OD*_0_ and *k* were considered accurate for a correlation coefficient R² > 0.95. However, one can observe a discrepancy at low optical densities, which could lead to the underestimation of low porous rocks. Table 2 and Fig. 5 presents the results from 2D analysis of both SPA and MPGDA as well as bulk porosities measured by TW method. For samples presenting anomalous areas, at least 2 areas (yellow dotted lines on Figs. 1,2) were used to ensure the representativeness of calculated porosities. Results from TW method give a range of porosity between 3.74 and 38.35%. Since no progress in fracture density is observed with increasing porosity, this wide range of porosities is mainly produced by the advancement of argillic alteration^36^. Porosities obtained from both autoradiography techniques were plotted with porosity results from TW method (Fig. 6). A linear regression shows a good correlation; R^2^ = 0.96 for SPA when compared to TW measurements, but only a fair correlation for MPGDA compared to TW (R^2^ = 0.78). The porosity values measured with different techniques are in fair agreement. When the comparison is made between MPGDA and SPA (Fig. 6c), linear regression shows a better correlation with the coefficient R^2^ is about 0.90 and a steepness of about 1. Considerable differences are found for samples with anomalous areas; possibly congruent with swelling clays. MPGDA porosities are slightly higher than the porosities measured by SPA which might be due to the difference between the linear ranges. Further development confirming the detector efficiency in MPGDA is in progress. It has to be noted here that the sample sizes in TW method are about 20 cm^3^ whereas the samples sizes in ^14^C-PMMA impregnation were about 200 cm^3^. Due to the heterogeneous structures of these samples the differences between TW and impregnation method are understandable.

**Table 2:** Porosity results for all samples from Triple-Weight method and from SPA and MPGDA.

Sample code	Triple Weight ϕ (%)	MPGDA ϕ (%)	SPA ϕ (%)
PA3	3.74 ± 1.11	6.37 ± 0.64	4.91 ± 0.49
PA4	7.33 ± 1.03	5.37 ± 0.54	3.9 ± 0.39
PA 7.2	7.2 ± 1.47	8.07 ± 0.81	5.79 ± 0.58
PA 10.1	13.84 ± 1.93	20.26 ± 2.03	14.86 ± 1.49
PA 11	38.35 ± 4.80	24.85 ± 2.49	31.69 ± 3.17
PA 12	17.05 ± 2.13	17.8 ± 1.78	17.36 ± 1.74
PA 13.1	10.5 ± 1.31	11.81 ± 1.18	11.04 ± 1.10
PA 13.2	4.48 ± 1.31	6.02 ± 0.60	4.27 ± 0.43

Over or underestimation of porosities is expected when a comparison is performed between 2D and 3D analyses of porous network of rocks.^37–40^ The porosities calculated from autoradiographs result from the 2D sections of samples while TW investigates all three dimensions of the porous network. The autoradiographs taken from 2D sections of samples could be non-representative of the sample’s total porosity. Notwithstanding these results and regarding the number of samples, the correlation should be supported with the measurements of additional clay-rich facies since only one sample (i.e. PA11) presents a high value of porosity. Porosities calculated from MPGDA are coherent with ones obtained from SPA.

From Geant4 simulation, the fraction *F*_*E*_of electrons reaching the sample surface is about 3.5% for a considered thickness of R*max* = 278 μm (R_*max*_ is the maximal range of E_*max*_ = 156 keV emitted beta particles in the PMMA). Thus, from the number of cpm mm^−2^ measured on the remains of resin, the efficiency of the detector is about 36%. This value is slightly lower than the one calculated by Samarati *et al*.^41^, which is about 50% for ^14^C, with another setup of PIM-MPGD device. The MPGDA based on PIM-MPGD device, has revealed to be an efficient technique for producing ^14^C-PMMA autoradiographs. The linear response of the PIM-MPGD device facilitates porosity calculations and produces reliable results, especially when a large range of porosities is investigated. SPA shows linear response in a range of activities depending on the exposure time, thus leading to tedious work on optimization of exposure time, whereas in the BeaQuant system the user is able, through the Beamage software, to control directly in real time the evolution of the activity on the autoradiograph during the acquisition. Therefore, since no potential saturation is possible (and provided that exposure time is sufficient) each acquisition performed can be subjected to analysis. Note however that the maximal activity supported by the acquisition electronic of the BeaQuant is 30000 cps.

In this study, porosity calculation depending of exposure time was not performed. Nevertheless, a new feature provided by the Beamage software will allow to produce different autoradiographs at different exposure times over the total duration of acquisition. It will be possible to follow properties such as porosity or fracture density depending on the exposure time and thus to determine appropriate exposure time for the property investigated. Regarding thin structures such as cleavages, it has been observed that the EA may require longer exposure times. This issue has to be studied depending on the nature and dimension of structures investigated.

## Conclusion

In this study, MPGDA technique using ^14^C-PMMA method for porosity determination and pore structure characterization was investigated on a series of rock samples showing hydrothermal alteration. The porosities of the samples were measured by the ^14^C-PMMA method using two different autoradiographic techniques for ^14^C detection; SPA and MPGDA. In addition porosities with TW method were compared to ^14^C-PMMA porosities. It has been shown that MPGDA provides similar results to SPA technique.

Moreover, the films are not needed and the detection limit of MPGDA is lower than in SPA.The straightforward measurement of ^14^C beta activity from the sample surfaces is huge advantage. In SPA the IP films are such that long exposures are not possible due to the autoerasing feature of the europium crystals in the films (“fading”). The low activities are not possible to detect by this method. However, low activities can be measured by MPGDA because there is no limit in acquisition time.

Procedure of porosity calculation is more straightforward than in SPA method. Good linear response and real time acquisition of the MPGDA could prevent over or under estimations. Regarding experiment conditions, the filmless nature of the technique as well as possible visual control during the acquisition are a substantial improvement compared to SPA. Artefacts due to dust particles in the sample surface during MPGDA acquisition have also been observed which is a slight drawback. These artefacts can however compromise large areas expected to be used in calculations and could not necessarily be detected at the beginning of the acquisition. The benefit in the MPGDA is that the background gamma is not disturbing the measurement at all whereas long exposures in SPA suffer from background gamma noise.

## Materials and Methods

### Rock samples

The comparative study presented in this manuscript has been conducted for a set of rocks originating from the Petite Anse-Diamant geothermal system. It corresponds to a retrograde system exhibiting, on its shallow part, an intense argillic alteration identified as the caprock of the fossil geothermal system^42^. The argillic alteration, with the formation of minerals such as pyrite and montmorillonite^43^, is expected to modify the petrophysical properties such as porosity of host rocks as shown in the work of Julia *et al*.^44^. In this work, 7 samples; from clay-poor to clay-rich facies were impregnated with ^14^C-PMMA in order to compare the two different autoradiographic methods for ^14^C activity detection and porosity calculation on a wide range of porosities. The samples were irregular in shape and the sizes varied from 200 to 400 cm^3^. The host rock of Petite Anse-Diamant geothermal system exhibits medium to fine-grained subautomorph crystals resulting from the volcanic activity of the Martinique Island. Its mineral composition reflects bulk chemical composition at the limit between andesite and dacite with characteristic minerals such as quartz^45^. In order to compare calculated porosities from both PSA and MPGDA methods to bulk connected porosity, water saturation of samples was performed in parallel using the TW method^46^.The degree of alteration of the studied rock samples is presented in detail in Delayre *et al*.^35^.

### ^14^C-PMMA method

The ^14^C-PMMA methodology has been developed by Hellmuth and Siitari-Kauppi^2,3^ and successfully applied in the field of rock porosity studies^7,8,15^. It allows notably a visualization of connected porosity represented as fractures and micro fractures, intergranular porosity and microporosity (matrix porosity, i.e. clay minerals) as well as quantitative calculation of the bulk porosity of samples. The methodology consists of impregnation of samples with ^14^C labelled MMA. After impregnation the MMA is then polymerized by irradiation or by heat with the chemical initiator fixing the radioactive resin into the porous network^4^. The MMA molecule has a low dynamic viscosity; 0.584 Pa.s for MMA compared with 1.005 Pa.s for water both at 20 °C allowing it to easily penetrate into the nanometer scale pore space of rocks. Moreover, due to its characteristics similar to water (such as the size of the molecule and dipolar character), MMA is able to enter in the interlayer space of clay minerals^47,48^ and thus makes it a suitable candidate for porosity studies dealing with altered rock samples. Due to the hydrophobic character of MMA, rock samples were first dried at 40 °C in the oven for at least one month to avoid insufficient impregnation due to the residual moisture in the samples. Then, the samples were impregnated with ^14^C-MMA with an activity of 178 kBq.mL^−1^ under vacuum for one month. The polymerization process was performed by placing the samples in a heat bath for 16 hours at a temperature of 55 °C. After the polymerization, each sample was sawed into two; a cut surface of 45 mm × 30 mm in size was polished to ensure optimal resolution in the ^14^C detection by SPA and MPGDA.

### Storage phosphor autoradiography

**Figure 7:**
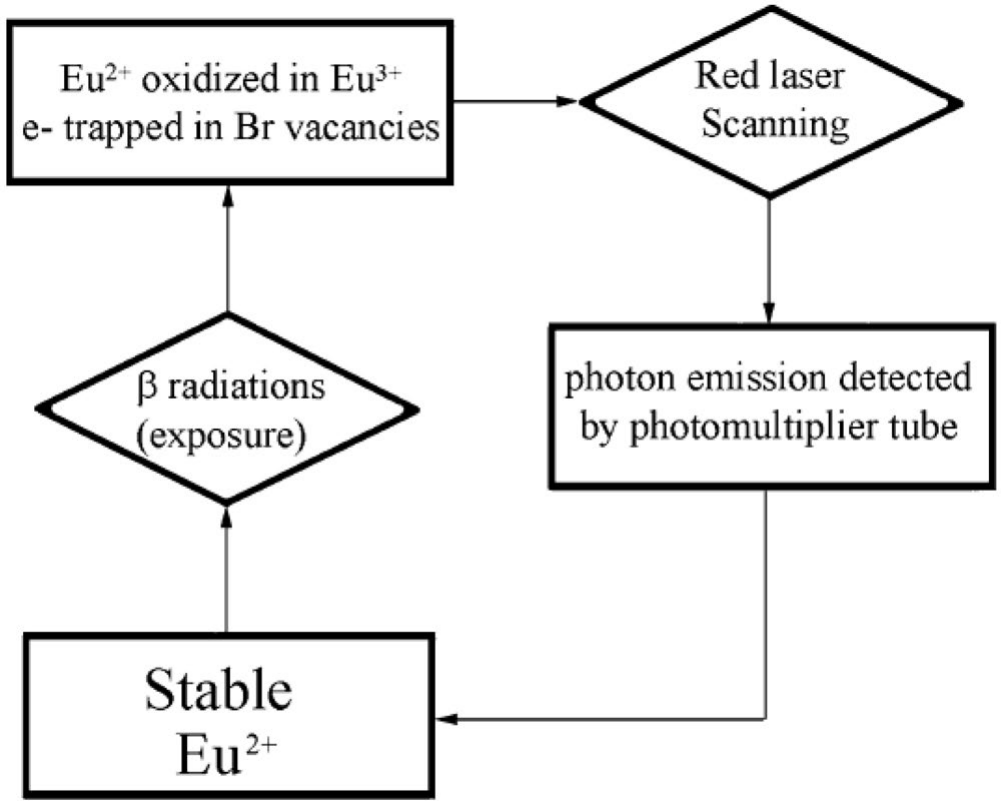
Digital photoluminescence process using phosphor IP for DA (Modified from L’Annunziata^14^).

IP plates used to perform SPA are usually made of three different layers: a protective layer (in direct contact with samples); a photostimulable layer and a support film. The photostimulable layer is generally composed of 5 μm crystals of BaFBr:Eu^2+^ embedded in an organic binder^1,14^. As pictured in the Fig. 7, under alpha and beta radiations, Eu^2+^ is oxidized to Eu^3+^. The resulting excited electron is then trapped in the F-center of BaFBr- complex. At this step, the latent image is stored on the IP. The stimulation of electrons trapped in BaFBr- complex with HeNe laser allows to liberate them and recombine with Eu^3+^. This leads to the emission of a photon at 390 nm while Eu^3+^ returns to its ground state^49^. The released photon is finally detected and multiplied by a photomultiplier tube and the spatial distribution of the ^14^C-PMMA can be computed as a digital image. After 3 days of exposure, the IPs were scanned using a FUJI-FLA 5000 digital scanner with a resolution of 1200 dpi and a 16-bit image depth to get the autoradiographs of the impregnated rock samples.

### Calibration and porosity calculation with SPA

A calibration of the phosphor screen is needed in order to convert the grey values of each pixel into porosity values. This has been performed using several PMMA standards of known ^14^C activities covering a range from 1 to 280 kBq.mL^−1^. In addition, the impregnation solution activity was taken from the ^14^C-PMMA around samples and have been used in the calculation of the porosities. Mean grey values for each standard have been extracted using the ImageJ software^50^ and corresponding optical densities have been calculated using the equation:

1 $$OD={\log }_{10}\left(\frac{I}{{I}_{0}}\right)$$

Where *OD* is the optical density; *I* the intensity of the pixel (grey value 0–65536) and *I*_0_ the mean intensity of the background measured on 5 different locations of the autoradiograph. In a previous work^15^, the authors observed a linear relationship in log-log scale between the activities and optical densities of standards. This linear shape can be modelled with the following power law equation:

2 $$OD=O{D}_{0}\cdot {A}^{k}$$

Where *OD*_0_ is the optical density for *A* = 1 Bq.mL^−1^; *A* the activity (Bq.mL^−1^) and *k* corresponding to the steepness of the model. From Eq. (2), the activity can be expressed as shown in equation:

3 $$A={\left[\frac{OD}{O{D}_{0}}\right]}^{\left(\frac{1}{k}\right)}$$

Depth penetration of beta particles produced from the radioactive decay of ^14^C is strongly influenced by the density of the matrix. Therefore, a beta absorption factor has to be added to take into account the difference of densities between the PMMA and rock described by equation:

4 $$\beta =\frac{{\rho }_{s}}{{\rho }_{0}}$$

Where *ρ*_*s*_ is the density of the sample and *ρ*_0_ the density of PMMA resin (*ρ*_0_ = 1.18 g.cm^−3^). Following Hellmuth *et al*.^2^, the density of the sample can be calculated with equation:

5 $${\rho }_{s}=(1-\varphi ){\rho }_{r}+\varphi {\rho }_{0}$$

*ρ*_*r*_ is the density for common minerals **(***ρr* = 2.7 g.cm^−3^). However, considering that *A*_0_ is the activity of the resin used for impregnation (*A*_0_ = 178 kBq.mL^−1^), the porosity (%) is merely calculated by:

6 $$\varphi =\beta \frac{A}{{A}_{0}}\cdot 100$$

In practice, one can then deduce a relation used for calculating the porosity (%) knowing the activity, combining Eqs. (4), (5) and (6):

7 $$\varphi =\frac{\frac{{\rho }_{r}}{{\rho }_{0}}}{1+\left(\frac{{\rho }_{r}}{{\rho }_{0}}-1\right)\frac{A}{{A}_{0}}}\frac{A}{{A}_{0}}\cdot 100$$

Once the porosity is calculated for each pixel of the autoradiograph, average porosity $\bar{\varphi }$ of the sample is computed from the sum of all porosities over the total number of pixels n of the area investigated, yielding to equation:

8 $$\bar{\varphi }=\frac{\sum \varphi }{n}$$

Uncertainties in the ^14^C-PMMA autoradiography arise from the physical parameters (grain density, tracer density, tracer activity), calibration parameters (minimum optical density, maximum optical density, fitting coefficient) and measured parameters; Grey level value of the background and grey level values obtained on autoradiograph^3^.

The mathematical expression for the total relative error is as follows:

9 $$\frac{\varDelta \varphi }{\varphi }=\frac{\varDelta {\rho }_{r}}{{\rho }_{r}}+\frac{\varDelta {\rho }_{0}}{{\rho }_{0}}+\frac{\varDelta {A}_{0}}{{A}_{0}}+\frac{\varDelta k}{k}+\frac{1}{B}\left(\frac{{D}_{0}}{{D}_{0}-D}\ast \frac{\varDelta {D}_{0}}{{D}_{0}}\right)+\frac{1}{B}\ast \frac{\varDelta D}{{D}_{0}-D}$$

where $\varDelta D=\frac{\varDelta {I}_{0}}{I}+\frac{\varDelta I}{I}$, φ is porosity and *k* is the fitting parameter. The calculated error is the maximum error for one pixel.

### The micro pattern gas detector autoradiography

**Figure 8:**
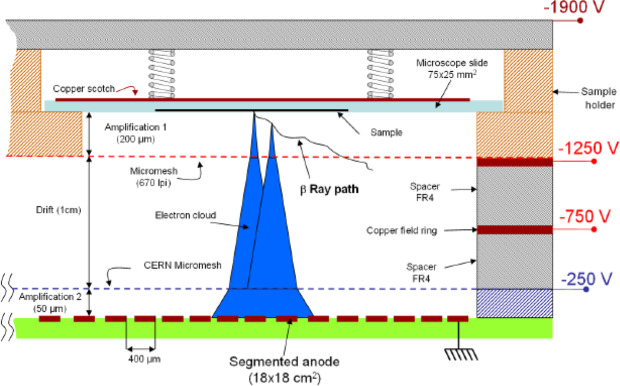
Principle of the PIM-MPGDA device^19^.

Electronic autoradiography is a filmless autoradiography technique operating with a parallel ionization multiplier (PIM) coupled with a micro pattern gas detector (MPGD)^19^. The technique is based on the acceleration of electrons, produced from the interaction between particles emitted from radioactive source and atoms of the gas mixture, by applying an electric potential. The ionization of the atoms of the gas mixture present between the cathode (the metallized surface of rock sample) and the anode yields to an amplification phenomenon by the production of an electron cloud which is eventually detected by the MPGDA. Thus, the PIM structure allows triggering an electronic signal for each electron cloud. As a result, MPGDA permits the real-time visualization of occurring disintegrations which makes it a very straightforward acquisition technique. Moreover, since the device does straight pulse counting, PIM-MPGD device offers a linear response over a large activity range (i.e. for the BeaQuant system: from 5 × 10^–4^ to about 900 cpm mm^-^²). The maximum counting rate is 30000 cps in the whole mapped area. Acquisition time will depend solely on the activity of samples (i.e. long acquisition time for low activities and vice versa). As seen in Fig. 8, the chamber filled with Ne + 10%CO2 gas mixture is delineated into three parts by Ni micromeshes^19^. Various types of sample holders have been developed for the BeaQuant enabling to investigate geological and biological microscope slides as well as entire rat slices (WBA)^19^. In this work, the sample holder customized for geologic samples (BELA) was used. Before starting the acquisition, one must carefully remove any possible dust particles present on the surface of samples, otherwise, artefacts may occur locally on the autoradiograph. Dust removing process can be performed by blowing clean compressed air as recommended by Donnard *et al*.^19^. The sample surfaces have to be polished carefully, and well cleaned; any roughness or dust particles on the surface cause artefact type signals on the autoradiograph. All samples were coated with a thin layer of conductive carbon to act as the cathode of the PIM system. In this work, the total acquisition time of MPGDA was 48 h.

### Porosity calculation with MPGDA

Since the PIM-MPGD device offers a linear response, the calibration is less complex as for SPA. It requires at least one standard of known activity which was provided by the remains of ^14^C PMMA resin around the samples. A series of 10 measurements were performed in the view to obtain reliable statistics. Following this step, a number of counts per minute and per mm² (cpm mm^2^) is calculated, which serves as reference for a porosity value of 100%. As for the calculation of porosity with SPA, beta absorption factor *β* from Eq. (4) is applied in the calculation:

10 $$\varphi =\frac{{N}_{s}}{{N}_{0}}\cdot \beta \cdot 100$$

where *Ns* and *No* are the number of cpm mm^−2^ for the sample and the MMA resin respectively.

### The Geant4 simulation for fraction of ^14^C beta emission reaching the detector

Geant4 (GEometry ANd Tracking 4) is a C^++^ based software toolkit, allowing to simulate the transport of charged particles, gammas and optical photons through the matter^51^. In the present study, beta particles are emitted from ^14^C-PMMA resin filling the sample porosity, but only a fraction of the emitted electrons can reach the sample surface and thus be detected by autoradiography. The passage of electrons emitted from ^14^C in PMMA resin has been simulated with Geant4, in order to predict the fraction of electrons reaching the sample surface^52^. This depends of the sample density and the range of ^14^C particles. Fractions of electrons reaching the sample surface have been calculated at different depths of sample up to R_*max*_ = 278 μm, which corresponds to the maximum range of electrons emitted by ^14^C in PMMA (i.e. R_*max*_ calculated here with Geant4 for the maximal emission energy of ^14^C, which is 156 keV). R_*max*_ is slightly higher to the one obtained by Kanaya-Okayama^53^ relationship (224 μm). An integral calculation has been achieved to estimate the average fraction of electrons reaching the surface sample, not from a single depth, but for a sample of thickness R_*max*_. Therefore, detector efficiency *ε* can be calculated using the following equation:

11 $$\varepsilon =\frac{{N}_{0}}{{N}_{a}\cdot {F}_{E}}\cdot 100$$

where *No* is the number of pulses (cpm mm^−2^) measured by MPGD from ^14^C-PMMA resin; *Na* is the total number of beta particles emitted per mm² and per minute in the volume of thickness Rmax, and was calculated from the activity of the ^14^C-PMMA resin. *F*_*E*_ [0–1] is the fraction of beta particles actually emitted up to the surface, and was determined by a Geant4 simulation run described above. The energy distribution of ^14^C used in the simulations come from the database provided in the Rad Toolbox toolkit developed by Oak Ridge National Laboratory^54^.Geant4.10.02.p02 version was used during this study, and a specific physics list has been constructed using the class *G4PhysicsListHelper*, which includes the standard electromagnetic processes/models. Simulations have been achieved with 10^6^ electrons for a good statistical analysis.

## Data Availability

The datasets analyzed during the current study are available from the corresponding author on reasonable request.
